# Molecular Dynamics and Docking Investigations of Several Zoanthamine-Type Marine Alkaloids as Matrix Metaloproteinase-1 Inhibitors

**Published:** 2017

**Authors:** Maryam Farrokhnia, Karim Mahnam

**Affiliations:** a *The Persian Gulf Marine-Medicine Biotechnology Research Center, The Persian Gulf Biomedical Sciences Research Institute, Bushehr University of Medical Sciences, Bushehr ,Iran.*; b *Biology Department, Faculty of Sciences, Shahrekord University, Shahrekord, Iran.*

**Keywords:** Matrix Metaloproteinase-1, Zoanthamine alkaloid class, Docking; Molecular dynamics simulation, MM-PB/GBSA

## Abstract

Zoanthamine-type alkaloids display a wide spectrum of biological effects. This study aimed to examine the inhibitory effects of norzoanthamine and its ten homologues of zoanthamine class on human fibroblast collagenase by modeling a three-dimensional structure of the ligands at collagenase using energy minimization, docking, molecular dynamics simulation and MM-PB/GBSA binding free energy calculations. The results showed that zoanthamide, zooxathellamine and enol-iminium form of norzoanthamine, with lower binding free energies than other compounds, are potent inhibitors of collagenase. However, the enol-iminium form of norzoanthamine showed a more inhibitory activity against collagenase than its keto form. This suggests that it can be used for treatment of many diseases such as osteoporosis, autoimmune diseases, and cancer. Zinc-binding residues such as His 118, His 122 and His 128 for hydrogen bonds and Leu 81, Tyr 110, Val 115, Leu 126, Pro 138, Ser 139 for hydrophobic interactions should be considered for designing an inhibitor for collagenase. Our theoretical results and MM/GBSA binding free energy calculations are consistent with experimental studies.

## Introduction

MMP-1(human fibroblast collagenase) is secreted by stromal fibroblasts, osteoblasts, and osteoclasts. It cleaves native collagen (abundant in bone matrix) at neutral pH (1). Overexpression of this enzyme is usually associated with the irreversible degradation of cartilage, tendon, and bone in people with arthritis (2). 

 Various types of mammalian collagenolytic enzymes are involved in the degradation of extracellular matrix including matrix metalloproteinases (MMPs), cathepsin K, and neutrophil elastase (3). The MMPs are members of a family of zinc-dependent proteolytic enzymes. They mostly appear key players in endochondral ossification during embryonic development, modeling, remodeling and homeostasis of the skeletal system (4, 5).

A cumulative evidence supports that MMP-1 cleavage activity is involved in many biological process such as keratinocyte migration and reepithelization, platelet aggregation, increased bioavailability of insulin-like growth factor 1 (IGF-1), cell proliferation, protease activated receptor-1 (PAR1) activation and pro-inflammatory and anti-inflammatory processes (for review, see reference 6 ). MMP-1 is also upregulated in tumor tissues and has been suggested to be associated with tumor invasion and metastasis (7). There is also a unifying link between MMP-1 and lung cancer risk (8). The MMP-1 significant contributions to these diverse biological and pathological conditions denote the probable involvement of this metalloproteinase in the pathogenesis of cancer and autoimmune diseases such as rheumatoid arthritis.

The active enzyme form of MMP-1 contains zinc- and calcium-binding catalytic domains with a COOH-terminal which may be involved in matrix binding. The structure is consisted of a twisted five-stranded beta sheet and three long helices. The collagenase active site cleft is bordered by a beta strand, a helix and a stretch of random coil adjacent to the COOH-terminus of that helix. The catalytic zinc is at the bottom of the cleft and is ligated by three conserved histidine residues, each at a distance of 2.1 Å. The second zinc ion is coordinated by two His and one Asp in an approximately tetrahedral manner. The calcium ion presented in the enzyme is ligated in an octahedral manner by two Asp and one Glu and two Gly and one Asn. Earlier studies demonstrated that zinc and calcium significantly stabilize the tertiary structure of collagenase (9).

Inhibition of MMP-1 activity may affect bone remodeling under certain pathological conditions. This can be related to the upregulation of interstitial metalloproteinases and bone loss. In order to selectively inhibit MMP-1 for therapeutic intervention, combined sophisticated theoretical and experimental approaches are required (10).

Studies on MMP inhibition, principally the structural determination of enzyme-inhibitor complexes, are crucial for understanding the structural features associated with inhibition. This information includes the scientific basis for a rational search for new and more potent inhibitors, through theoretical chemistry calculations (11, 12, 13).

Traditionally, the design of an MMP inhibitor (MMPi) has involved the use of zinc-binding groups due to the fact that MMP cleavage on substrates directly involves the catalytic Zn^2+^ ion(s) (14, 15, 16). Upon binding of the substrate, the zinc-bound water molecule attacks the substrate carbonyl carbon atom and the transfer of protons through a preserved Glu residue to the amide nitrogen of the scissile bond results in peptide cleavage (9). Therefore, zinc-binding groups (ZBG) in MMPi displace the zinc-bound water molecule and inactivate the enzyme (11). In addition, the ZBG acts as an anchor to lock the MMPi in the active site and directs the inhibitor into the target substrate-binding pockets.

As one of the members of zoanthamine class of marine alkaloids, there exist high concentrations of norzoanthamine in the epidermal tissues of *Zoanthussp* (17). Over the past 20 years, more than twelve nitrogenous marine metabolites known as zoanthamine alkaloids have been reported. They show a wide spectrum of biological effects including the inhibition of phorbolmyristate acetate-induced inflammation in mouse ear (18); inhibition of the growth of P-388 murine leukemia cell lines (19); inhibitory effects on thrombin-, collagen-, and arachidonic acid-induced human platelet aggregation (20); suppressive effects on interleukin-6 production (21); anti-osteoporosis activity in ovariectomized mice (22); acceleration in the formation of the type I collagen-hydroxyapatite composites (23); enhancement of collagen release from the immobilized matrix vesicle model and suppression of the proteolysis of bovine serum albumin, elastin and collagen (23).

Moreover, it has been suggested that collagen-norzoanthamine supramolecular association may be the mode of anti-osteoporosis action of this marine alkaloid (23). The collagen protective activity of norzoanthamine suggests that these marine alkaloids may be a promising drug candidate for the prevention or treatment of osteoporosis-associated bone loss. In the present study, we examined the inhibitory effects of norzoanthamine and its homologues on human fibroblast collagenase by using theoretical chemistry studies.

*Methods and Computational Details*

**Figure 1:**
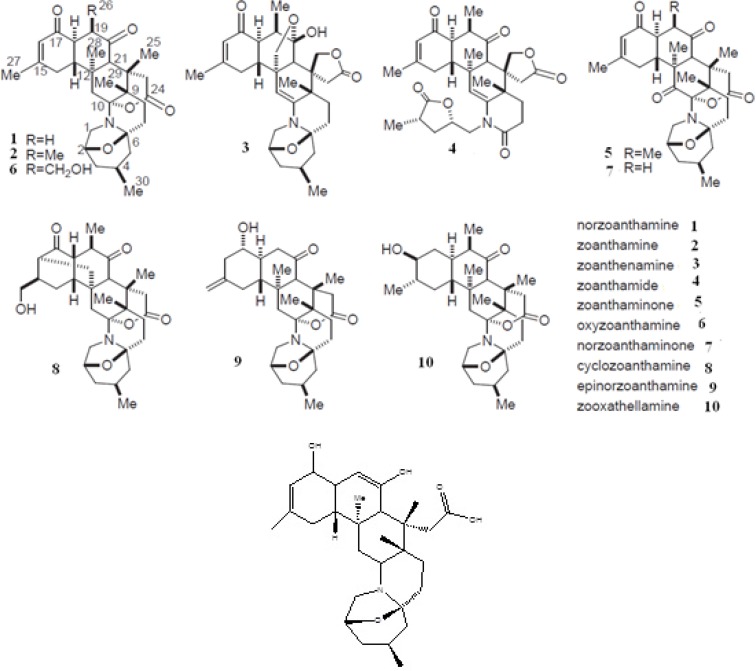
Structure of norzoanthamine and its homologues (A) and enol-iminium tautomer of norzoanthamine (B). The relative stereochemistry of molecules has been determined by an advances version of Mosher's method

The crystal structure of fibroblast collagenase was extracted from PDB bank (PDB code: 1CGL) (9). This structure refers to collagenase co-crystallized with an inhibitor (N-[(1s)-3-{[(benzyloxy) carbonyl] amino}-1-carboxypropyl]-l-leucyl-N-(2-morpholin-4-ylethyl)-l-phenylalaninamide) (OED). Since PDB structure has two identical chains and collagenase has only one chain, the chain A of protein was just retained for this study. 2D structures of the studied ligands (molecules 1 to 10 and the molecule1 in its enol-imine tautomer known as ligand 1-enol), were drawn (Figure1) and minimized via Hyperchem 8 software (24). They were then optimized at the density functional theory (DFT) level. The B3LYP hybrid functional was applied to these systems (25, 26). For electronic and structural investigations of ligands, the 6-31+G* basis set was considered. Finally, the vibrational frequencies were computed to elucidate the global minimum structure. All calculations were performed using the Gaussian 2003 program (27). The binding affinities between the ligands and the active sites of the enzyme were studied using docking studies (AutoDock 4.2 suites, 28). MD simulation and MM-PB/GBSA binding free energy calculations were performed via Amber package version 12 (29). All calculations were repeated with a crystallographic ligand (OED) as control. Afterwards, eleven ligands were studied.

*Molecular docking*

All ligands plus the co-crystallized inhibitor (OED) were docked into the chain A of original PDB file. The estimated binding affinities between the ligands and the active sites of the enzyme were then explored by docking studies (AutoDock 4.2 suites) (28). Input files required for AutoDock 4.2 were made in Autodock Tools (ADT) (Autodock Tools, 3.0). The Gasteiger charges were assigned to the ligands. All files were converted to the PDBQT format needed for Autodock calculations. The protein and ligand were considered rigid and flexible, respectively during docking procedure. 

At first, docking calculations were carried out with performing a blind docking. The grid box was centered on the original ligand position of the crystal structure (OED). Then the lowest docking energy of each ligand was selected. Further docking calculations were done to obtain the top-ranked pose. All hydrogen atoms were added to the collagenase protein and after assigning Kollman united atom charges and atomic solvation parameters, then the non-polar hydrogens merged. The size of the grid box for final docking to specify the search space was set at 60×60×60, and centered on the predicted cavities with a default grid point spacing of 0.375 Å. Pre-calculated grid maps storing interaction energy of the ligand atom type with the receptor target, were obtained using AutoGrid4. 

The zinc parameters used in this study were radius(r = 1.1Å) and well depth (ε = 0.25 Kcal/mol) (31). A charge of +2.0 e was assigned to the zinc and calcium ions (31) in PDBQT file.

The Lamarckian Genetic Algorithm (LGA) (28) was used for a conformational search approach with 200 runs of Genetic Algorithm runs. A maximum number of 2,500,000 energy evaluations per run were carried out, while crossover and mutation rates were run with the default parameter settings. The step size parameters were changed, i.e. "Translation" to 0.2 Å, "Quaternion" to 5.0°, and "Torsion" to 5.0°.

AutoDock4.2 applied to evaluate the binding free energy was used to discover the best binding mode. The energy scoring values of AutoDock4.2 are consisted of intermolecular energy (van der Waals, hydrogen bonding, desolvation and electrostatic energies), total internal energy and torsional free energy. In fact the lower the binding free energies, the more effective the ligand-binding protein. The root-mean-square deviations of ligands (RMSD) were calculated in order to put them into families of similar conformations or "clusters". Therefore, the expected docked complexes are preferred based on the lowest binding free energy from the largest"clusters" for each ligand.

The docked conformations of each ligand were ranked into clusters based on the binding energy. The results were clustered with the tolerance of 2.0 Å RMSD. Based on the lowest binding free energy of the most populated cluster, the best position of each ligand was chosen for subsequent studies.

*Molecular dynamics (MD) simulations*

The best docked complexes were selected for MD simulations. Twelve MD simulations (11 ligand and OED) were performed using the Amber12 package (29). The protein was modeled using the AMBER FF99SB force field. The inhibitor charges were obtained using the RESP fitting procedures (32). Topology prep files for ligands were built with the general amber force field (gaff) using Antechamber module in AmberTools12 (33). The complexes were then solvated in a truncated octahedral box of TIP3P (34) water molecules with a buffer size of 12 Å, and neutralized using 10 Na^+^ ions. The default protonation of Amber12 was used for titratable residues in protein.The energy minimization and MD simulations were performed for each system using the SANDER module of the Amber12 package (29).First, 10000 cycles of minimization (MAXCYC) with the first 700 steps of steepest descent (NCYC) and 9300 steps of conjugate gradients on all atoms of the system were performed to relieve the bad steric interactions and to get closer to an energy minimum. After energy minimization, the position restraints at constant volume (NVT) for 100 ps, with a restraint force of 10 Kcal/mol at temperature of 100 K, and then at constant pressure (NPT) for 100 ps, with a restraint force of 1 Kcal/mol at temperature of 300 K, were performed. 

Next, an equilibration step at constant pressure (NPT) at 300 K was performed. The duration time in this step was 100 ps and the time step was 2 fs, while the restraint force was removed in this step. This approach allows the water to equilibrate around the protein and come to an equilibrium density. In the position restraints and equilibration steps, Langevin dynamics were used to control temperature.

Finally, a production step for 10 ns with a 2 fs time step at constant pressure (NPT ensemble) and with an isotropic position scaling (ntp = 1) at 300 K was carried out. In this step, the Berendsen thermostat was used to control temperature (ntt = 1). All bonds with hydrogen atoms in all steps were constrained via the SHAKE algorithm (35) (ntc = 2, ntf = 2). The periodic boundary was used in all steps (ntb = 1). The long-range electrostatic interactions were treated using the particle mesh Ewald method (36), and a nonbonded cutoff was set to 10 Å. The zinc and calcium ions were connected to related residues via tleap module of Amber12. The images of ligands in the active site of protein were generated with LigPlus (37).

*MM-PB/GBSA calculations*

Molecular mechanics Poisson Boltzmann/surface area (MM-PBSA) is regarded as an attractive approach for drug design since it works not only for small and medium size organic compounds but also for the biological molecules such as proteins and DNA. The MM/PBSA is a molecular dynamics based approach that is fast and practical for binding free energy calculations, in spite of free energy perturbation (FEP) or thermodynamics integration (TI) that are time-consuming. Also, unlike the linear interaction energy (LIE) method, MM-PBSA applies no empirical parameters in its free energy calculations. It does not need a training set to derive the empirical parameters as does LIE, which makes it a promising method for ranking very different compounds from database searching for binding to a given site (38). Therefore, by combining this method with molecular docking and molecular dynamics simulations, one can hope to reliably model a protein and DNA complex (38).

The binding energies in this study were calculated by the MM-PBSA method as implemented in Amber 12 (39-43). Four hundred snapshots were extracted from MD simulation trajectories for binding free energy calculations. The water molecules and counter-ions were stripped.

In summary, the binding free energy is:

Eq(1) $$∆G_{\mathrm{binding}}=G_{\mathrm{complex}}-\left[G_{\mathrm{protein}}+G_{\mathrm{ligand}}\right]$$

Each free energy term in Eq (1) is computed using Eq(2):

Eq(2) $$G=E_{\mathrm{gas}}+G_{\mathrm{solvation}}-T∆S$$

The entropy term is written as T∆S. ∆E_gas_ stands for the gas phase or vacuum molecular mechanical energy and can be divided into the van der Waals, electrostatic, and the internal energy contributions in gas phase. ∆G_solvation_ is the solvation free energy and estimated using continuum solvent methods. It can be partitioned into two parts: the polar contribution (∆G_(PB/GB)_) and the nonpolar contribution (∆G_nonpolar_) as shown in Eq(3).

Eq(3) $$∆G_{\mathrm{solvation}}=∆G_{\mathrm{PB}/\mathrm{GB}}+∆G_{\mathrm{nonpolar}}$$

The electrostatic solvation free energy was determined using the finite difference PB or GB models. The nonpolar contribution to the solvation free energy was calculated from the solvent accessible surface-area.

Eq(4) $$∆G_{\mathrm{nonpolar}}=\gamma \times ∆\mathrm{SASA}+b$$

Where ∆SASA (solvent accessible surface-area) was determined with linear combinations of pairwise overlaps (LCPO) method (44). The corresponding solvation parameters γ and b are 0.00542 Kcal/molÅ^2^ and 0.92 Kcal/mol, respectively for the PB method, and 0.0072 Kcal/molÅ^2^ and 0 Kcal/mol respectively, for the GB method. The probe radius of the solvent was set to 1.4 Å. PARSE radii were used in solvation model (45). The obtained conformational entropy change upon ligand binding (T∆S) from the sum of the translational, rotational and vibrational components, was computed using quasi-harmonic entropy approximation. In this study, the single trajectory approach was applied to estimate the energies. This means that the protein and ligand geometries were taken from protein-ligand complex, thus there is no internal energy contribution to the net molecular-mechanical binding energy (∆E_gas_). Estimation of energies in this manner has been proven successful in many studies (46). The separate trajectory approaches in which three trajectories of complex, free receptor and free ligand used for energy calculations were deficient in practice due to the limitations of sampling and large fluctuations. 

## Results

The results of the computations performed in the present work were divided into three sections according to the applied computational techniques for ligands studies. All vibrational frequencies of the ligands were positive (data not shown), suggesting the optimized structures from all ligands had been in the global minimum of the potential energy surfaces. All of the docking modes were near catalytic zinc ion (Zn 169) in the active site of the enzyme. The second column of Table 1. shows the electronic energy for all optimized geometries of ligands at B3LYP/6-31+G* level of theory. The third column shows the van der Waals and hydrogen bonds plus desolvation energy components of estimated docking energies. Electrostatic interactions and the lowest binding free energy of each ligand in the active site of collagenase have been shown in the 4^th^ and 5^th ^columns, respectively. The results of top-ranked docking pose conformers of the ligand 1 in its keto and enol-iminium are interesting. As seen in Table 1. the enol-iminium form of norzoanthamine shows a much more inhibitory effect than its keto form and its estimated binding energy is lower than all ligands. The enol-iminium tautomer of norzoanthamine was investigated as a model of norzomanthamin hydrochloride.

*Molecular dynamics simulations*

Based on the initial structures of the systems from the molecular docking, MD simulations were performed to observe the dynamic behavior of the investigated ligands in the active site of collagenase and to check the stability of the docked complexes. To evaluate the quality of MD simulations, the structural properties of the receptor–ligand complexes were monitored during the MD trajectories of each system.

**Figure 2:**
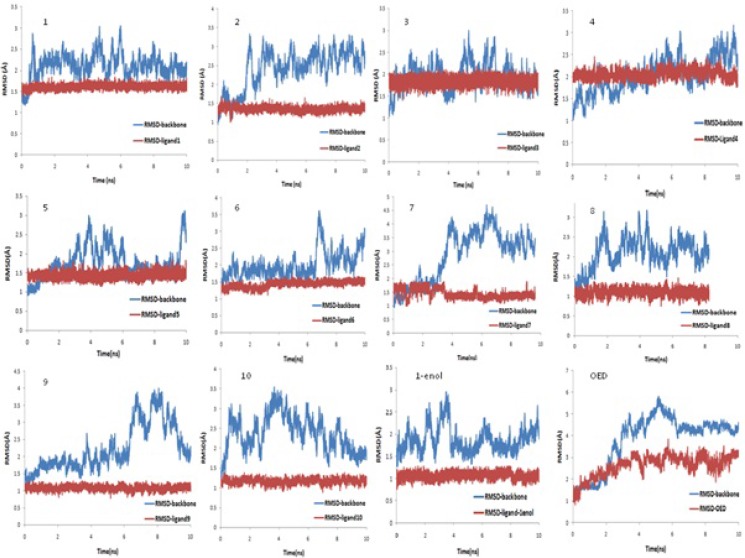
RMSD of protein backbone and RMSD of ligands during 10 ns MD simulation

Figure 2. shows the RMSDs of ligand atoms and the protein backbone atoms against their initial positions. The average RMSD values of the last 4 ns of 10 ns MD simulation for backbone atoms of the proteins in addition to all atoms of the ligands plus zinc and calcium atoms, relative to their initial positions were displayed in Table 2. As seen, the protein and ligand structures are relatively stable in all of the twelve simulation systems (11 norzoanthamine homologue and OED). These results show that the simulations were stable with a maximum average RMSD of about 4.3 Å for protein backbone in complex with OED. The average RMSD of protein backbone in complex with other ligands was about 2 Å (Table 2). In general, the systems followed the same trend in all cases during the simulations, and the RMSDs tended to converge in the early phase of the simulations.

The position of zinc and calcium ions were stable during the MD simulations as the average maximum RMSDs of these atoms is about 1.3 Å with a trivial standard deviation. For temperature and energy conservations, a slight fluctuation was observed in all of the systems during the first 1 ns of simulation then flattened out for standard deviations of less than 1.5 K for the temperature and about 90 Kcal/mol for potential energy (Table 3). Minor changes in standard deviation of temperature, potential and kinetic, total energies and density during 10 ns MD simulations (Table 3) show that the length of MD simulation was adequate for production step and that simulations were stable under simulation conditions.

**Figure 3:**
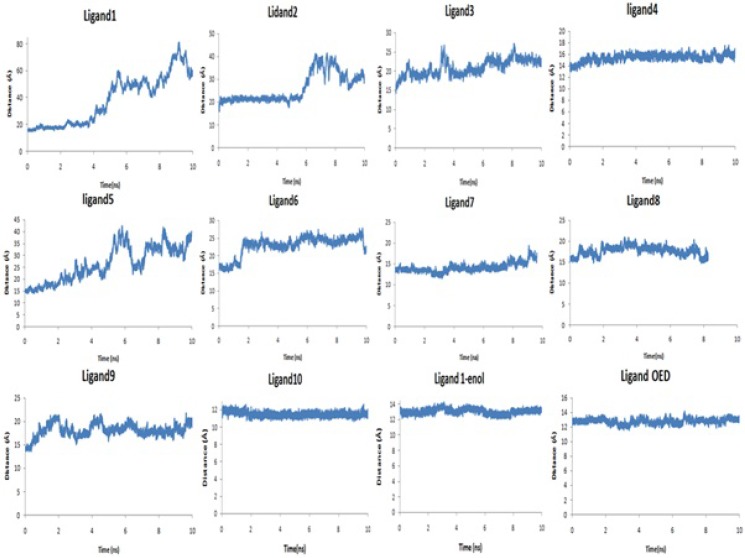
Distance (Å) between center of mass of collagenase and ligands during 10 ns MD simulation

Figure 3. and Table 2. show the distance between the centers of mass of protein and ligands during 10 ns MD simulation. These results and the visual inspection of final structures indicate that ligand 4, 10, 1-enol and OED reside in the active site of the enzyme, but other ligands go away from active site (data not shown). This finding was confirmed with snapshot with the lowest potential energy across the simulation.

**Figure 4:**
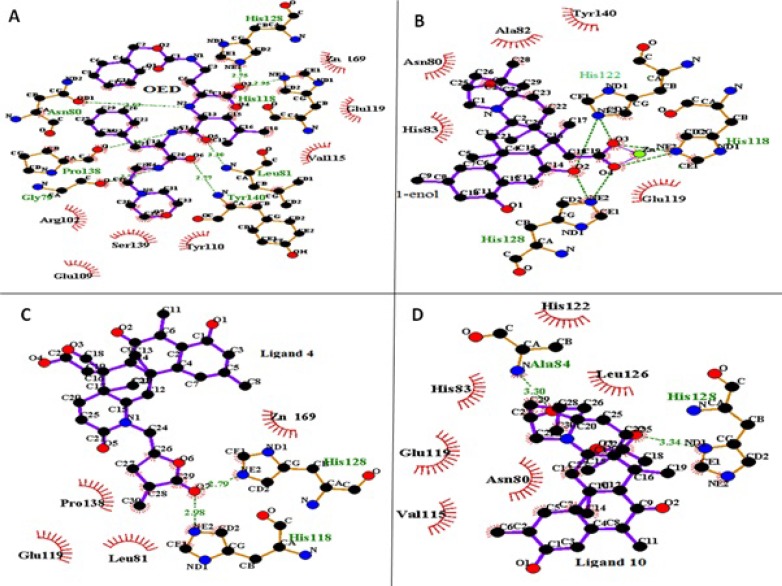
LigPlus pictures of ligand OED (A), ligand 1-enol (B), ligand 4 (C) and ligand 10 (D) in the active site of collagenase after 10 ns MD simulation. Hydrogen bonds were shown with green dotted line. Hydrophobic interactions were shown in thicker lines

Figure 4. shows LigPlus (37) presentation of ligand OED, 1-enol, 4 and 10 in the active site of collagenase after 10 ns MD simulation (final structures). In complex of ligand OED with the protein Gly 79, Asn 80, Leu 81, His 118, His 128, Pro138, Tyr 140 residues make hydrogen bonds with OED. The analysis of complex trajectories shows that Asn 80 and Glu 119 have hydrogen bonds with OED ligand at the major part of simulation time. In addition, Tyr 110 and Val 115, Ser 139 have hydrophobic interactions with ligand OED (Figure 4A). The enol-iminium form of norzoanthamine (1-enol) has interactions with catalytic zinc ions; while this interaction is absent in its keto form The carboxyl group of 1-enol ligand (O3 and O4 in Figure 4B) with the negative charge makes a salt bridge with catalytic zinc ion with the positive charge. Some hydrogen bonds were observed between the NE2 atoms of His 118, 122, 128 and hydroxyl and carboxyl groups of ligand 1-enol (O2, O3 and O4 at Figure 4B). Also, the analysis of complex trajectories of 1-enol shows that Glu 119 has hydrogen bonds with ligand 1-enol at the major part of simulation time. In addition, hydrophobic interactions of Ala 82, His 83, and Tyr140 were detected with aza-bicyclo octane tail of ligand 1-enol, orientating deeply into the Zn169 pocket of collagenase. In ligand 4, ketonic oxygen (O7 in Figure 4C) of lactone (tetrahydropyran) tail makes hydrogen bond with NE2 atoms of His 118 and His 128 and lactone tail has hydrophobic interactions with Pro 138 and Leu 81 (Figure 4C). In ligand 10, ketonic oxygen of lactone makes hydrogen bond with ND1 atom of His 128. Also, oxygen of aza-bicyclooctane makes hydrogen bond with amide nitrogen of Ala 84. His 83, Val 115 and Leu 126 have hydrophobic interactions with ligand 10, which stabilizes the complex (Figure 4D). Finally, ligand 1-enol, 4 and 10 have similar hydrogen bonds with crystallographic ligand (OED).

*Binding free energy calculations*

In order to explore the inhibition strength of all investigated ligands with collagenase active site, the MM-GB/PBSA method (47) was used. This method calculates the binding free energies of ligands 1 through 10, Also ligand 1 in its enol-iminium tautomer and OED with collagenase (Table 4). Without considering OED ligand, the lowest binding free energy belongs to ligand 1-enol. However, for most complexes investigated, the contributions of the entropy changes (TΔS) to the free energies almost weakened the binding of inhibitors to collagenase.

It has been shown in earlier studies that neglecting the entropy effects of a set of analogues ligand binding to the same receptor results in good agreement between the calculated and experimental relative binding free energies (46). Similarly, in the present study, entropy contribution (T∆S) changed from -46 to -80 Kcal/mol at 298.15 K (except ligand 1-enol) (Table 4). Consequently, the entropy contributions were indeed neglected as we are only interested in the relative order of binding affinities and there was no experimental binding free energy of these ligands to the collagenase.

Without considering entropy effects, the ΔG_binding_, calculated for all ligands ranged from −595 to 71 Kcal/mol, based on the Generalized Born method. Ligand 1-enol had the most negative ΔG_binding_ value (−201.37 Kcal/mol for MM-GBSA) and ligand 5 had the largest binding free energy value (71.42 Kcal/mol) (Table 4). Interestingly, there was a positive regression between docking energy and Generalized Born binding energy with and without entropy as in both cases; the regression coefficient between them was about 41 percent. On the other hand, there was no regression between docking and Poisson-Boltszmann/surface area binding energy (R^2 ^= 0.07). According to the energy components of the binding free energies listed in Table 4. both electrostatic (∆E^elec^+∆G^GB^) and van der Waals plus hydrophobic (∆E^vdw^+∆G^SA^) energies are important for ligand 4 and OED. In ligand 4, electrostatic interactions between ketonic oxygen of lactone ring and zinc ion keep this ligand in the active site of the enzyme.

**Figure 5:**
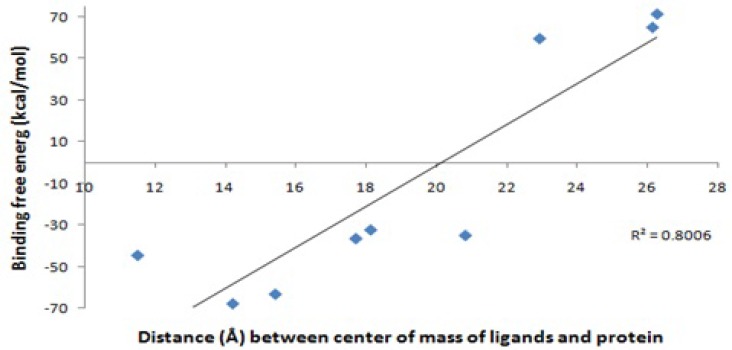
Distance of center of mass ligands and protein (Å) against Generalized Born binding free energy (∆GGB) (Kcal/mol) without entropy

In the case of the ligand 1-enol, contribution of electrostatic interactions (∆E^elec^+∆G^GB^) in binding free energy is more important than van der Waals plus hydrophobic interactions (∆E^vdw^+∆G^SA^). In other words, in the ligand 1-enol, the salt bridge between carboxyl oxygen and zinc ion is favorable, but van der Waals and hydrophobic interactions of Ala 82, His 83, Tyr 140 with aza-bicyclo octane group are not very well with unfavorable contribution. In binding ligand 10, van der Waals and hydrophobic interactions are the major favorable contributors of binding. This finding suggests that the interaction mechanisms of these ligands may differ from the others. These results are consistence with docking results (Table 1). There is a positive regression between distance of the mass centers of ligands and protein (Table 2) and Generalized Born binding free energies without entropy (Table 4).Without consideration of the ligand 1, 1-enol and OED (as outliers), the regression coefficient between them is about 80 percent. Figure 5 shows that an analogy can be seen between MD simulations and binding free energy calculations. It seems that in the current study the Generalized Born method has been more applicable than Poisson-Boltszmann/surface area one.

## Discussion

Based on the obtained results of the molecular docking, molecular dynamics simulation, and MM/GBSA binding free energy ligand 1-enol, ligand 4 (zoanthamide), and ligand 10 (zooxathellamine) stay in the active site of enzyme during 10 ns MD simulation and show higher inhibitory effects towards collagenase relative to other norzoanthamine derivatives. These compounds are able to interact with the active pocket of collagenase by several hydrogen bonds, hydrophobic contacts, and make a salt bridge with catalytic zinc with negative Generalized Born binding free energy. In addition, the ligand 1-enol is better inhibitor than the ligands 4 and 10. The ligand1-enol has significant inhibitory effects on MMP-1 through interactions with the catalytic zinc. The results show that enol-iminium form of norzoanthamine (as a model to norzoanthamine hydrochloride) interacts with the catalytic zinc however the keto form of norzoanthamine does not. On the other hand, Yamaguchi (22) have reported *in-vivo* anti-osteoporosis activity of norzoanthamine hydrochloride through experimental studies. They showed that the oral administration of norzoanthamine hydrochloride completely suppressed the loss of trabecular bone, and thickened the cortical bone in ovariectomized mice (22). Theoretical results of the present study showed that the inhibitory effect of the ligand 1-enol was more than ligand 1. Therefore, it can be claimed that a possible mechanism of norzoanthamine hydrochloride action may be through inhibition of collagenase I. The development of synthetic and natural MMP-1 inhibitors such as the ligand 1-enol, promises for not only novel therapeutic measures for the prevention and treatment of osteoporosis, but it will also provide potential therapeutics for the modulation of extracellular matrix remodeling, and the disturbances of the microenvironment involved in the pathogenesis and disease progression of some cancers and autoimmune diseases.

**Table 1:** Electronic energy and electrostatic and van der Waals energies and the lowest binding energy from docking results

**Molecule**	**E**_electronc+ZPVE_^1^	**vdw+hbond +desolvation Energy**	**Electrostatic Energy**	**ΔG**_binding_^2^
Ligand 1-keto	-978081	-7.13	+0.04	-7.09
Ligand1-enol	-978031	-5.39	-8.20	-12.09
Ligand 2	-1002726	-7.28	-0.04	-7.32
Ligand 3	-1095966	-8.49	-0.21	-8.40
Ligand 4	-1096901	-8.72	+0.08	-8.05
Ligand 5	-1049180	-7.81	-0.11	-7.92
Ligand 6	-1049920	-8.84	0.05	-8.30
Ligand 7	-1024538	-7.57	0	-7.56
Ligand 8	-1019399	-6.04	-1.27	-6.72
Ligand 9	-978473	-7.35	-0.09	-7.14
Ligand 10	-1004747	-7.62	-0.09	-7.42
OED	-1309458	-9.31	-6.79	-9.54

1 Electronic energy (kcal/mol).

2 The lowest binding free energy from docking calculations (kcal/mol).

**Table 2:** The average and standard deviations of RMSD (Å) of protein backbone and ligand atoms and also RMSD of zinc and calcium ions at the last 4 ns of 10 ns MD simulation. In addition, distance (Å) between center of mass ligands and protein during 10 ns MD simulation was mentioned

	**RMSD of protein backbone**	**RMSD zinc and calcium ions**	**RMSD of ligands**	**Distance **
Ligand 1-keto	2.08±0.19	1.02±0.11	1.62±0.05	38.16±18.35
Ligand 1-enol	1.87±0.2	1.05±0.1	1.06±0.07	13±0.34
Ligand 2	2.73±0.24	1.05±0.22	1.35±0.06	26.13±6.35
Ligand 3	1.97±0.28	0.53±0.12	1.85±0.1	20.81±2.09
Ligand 4	2.3±0.3	1.32±0.3	2.1±0.1	15.41±0.65
Ligand 5	1.7±0.37	0.8±0.1	1.45±0.1	26.26±7.4
Ligand 6	2.3±0.4	1.01±0.14	1.5±0.05	22.92±2.74
Ligand 7	3.5±0.44	0.94±0.1	1.35±0.1	14.2±1.12
Ligand 8	2.2±0.2	0.93±0.25	1.08±0.1	17.7±1.07
Ligand 9	2.9±0.56	1.3±0.25	1.06±0.07	18.12±1.4
Ligand 10	2.15±0.33	0.81±0.11	1.18±0.06	11.5±0.27
OED	4.3±0.2	1.04±0.14	2.88±0.26	12.81±0.34

**Table 3:** The average and standard deviations of temperature (K) of kinetic (EKCMT ) and potential (EPTOT) and total (ETOT) energies (kcal/mol) and density (gr/cm^3^) during 10 ns MD simulation for all complexes

	**Temperature **	**EKCMT**	**EPTOT**	**ETOT**	**Density**
Ligand 1-keto	299.96± 1.48	6481± 56	-75284± 4	-60449± 39	1.024± 0.001
Ligand 1-enol	299.96±1.49	6480± 57	-75210±83	-60370± 37	1.024± 0.001
Ligand 2	299.96± 1.49	6469± 57	-75147± 84	-60329± 39	1.024± 0.001
Ligand 3	299.96±1.49	6478± 57	-75272± 5	-60437± 41	1.024± 0.001
Ligand 4	299.96± 1.49	6481± 57	-75858±85	-61020± 42	1.025± 0.001
Ligand 5	299.96±1.49	6471± 57	-75179±83	-60360± 38	1.024± 0.001
Ligand 6	299.96±1.49	6477± 57	-75245± 84	-60412±39	1.024± 0.001
Ligand 7	299.96 ±1.49	6475 ±57	-75276±89	-60450± 6	1.024 ±0.001
Ligand 8	299.97 ±1.48	6467 ±57	-75163±83	-60351± 40	1.024 ±0.001
Ligand 9	299.96± 1.49	6480± 57	-75271±83	-60435± 38	1.024± 0.001
Ligand 10	299.96±1.48	6470± 57	-75110±86	-60289± 43	1.023± 0.001
OED	299.96±1.48	6501± 57	-75738± 89	-60847± 49	1.024± 0.001

**Table 4:** The Generalized Born (∆G_GB_) and Poisson-Boltszmann (∆G_PB_) binding free energy and electrostatic and hydrophobic and entropy contribution (T∆S) in free energy binding in Kcal/mole at 298.15 K

**molecule**	**∆G**_GB_^a^	**∆G**_GB_^b^	**∆G**_PB_^a^	**∆G**_PB_^b^	**∆E** ^elec^ **+∆G** ^GB^	**∆E** ^elec^ **+∆G** ^PB^	**∆E** ^vdw^ **+∆G** ^SA^	**T∆S**
Ligand 1-keto	-19.8	26.26	-4.06	42	5.01	3.17	34.15	-46.06
Ligand 1-enol	-201.37	-141.44	-32.40	27.54	-155.2	-34.36	32.02	-59.93
Ligand 2	65.04	118.88	88.46	142.3	5.32	4.24	29.69	-53.83
Ligand 3	-34.80	20.41	-4.28	50.93	10.01	9.17	22.30	-55.21
Ligand 4	-62.99	17.82	29.82	110.63	-45.07	15.48	-17.92	-80.81
Ligand 5	71.42	129.99	88.63	147.20	4.40	4.03	36.28	-58.57
Ligand 6	59.67	128.02	90.82	159.18	9.29	9.51	24.06	-68.36
Ligand 7	-67.58	-1.25	9.78	76.11	18.77	47.52	10.25	-66.33
Ligand 8	-36.33	23.90	6.01	66.25	7.79	13.19	18.35	-60.23
Ligand 9	-32.16	24.2	5.34	61.69	12.49	11.08	16.96	-56.35
Ligand 10	-44.38	21.6	15.71	81.7	24.68	24.39	-4.18	-65.99
OED	-595.96	-481.17	-447.82	-333.03	-63.77	7.66	-12.61	-114.8

a: Without entropy approximation ($∆E_{\mathrm{gas}}+∆G_{\mathrm{solvation}})$. b: Using quasi-harmonic entropy approximation ($∆E_{\mathrm{gas}}+∆G_{\mathrm{solvation}}-T∆S$).

The mode of the anti-osteoporosis effects of norzoanthamine (ligand 1) was heavily investigated by Kinugawa *et al*. and some other workers (23). They found that norzoanthamine protects collagen from proteolytic cleavage and stabilizes its secondary structure. Recently, Genji (17) showed that norzoanthamine probably protects collagen by increasing its resistance to UV light. Norzoanthamine inhibits collagenase activity against type I collagen (17, 23). It has been suggested that norzoanthamine has no interaction with the active sites of collagenase, but it makes a non-covalent interaction with collagen, protecting it from proteolytic cleavage (23). Our theoretical results are consistence with above experimental results and show that norzoanthamine (ligand 1) could not stay in the active site of collagenase and that its binding free energy is positive.It is noteworthy to mention that in future theoretical studies, it would be interesting to investigate the interaction of norzoanthamine with collagen. Different modifications of norzoanthamine may improve its interaction with collagen.Therefore, based on the obtained results from Table 4 and Figure 4. it can be concluded that a good inhibitor of collagenase makes a salt bridge with zinc ion 169 and hydrogen bonds with His 118, His 122, His 128 and hydrophobic interactions with Leu 81, Tyr 110, Val 115, Leu 126, Pro 138, Ser 139.

## Conclusions

Some of nitrogen marine metabolites known as zoanthamine alkaloids act as robust inhibitors of MMP-1. Theoretical and experimental studies are consistent in the case of OED ligand and norzoanthamine and its enol-iminium form of norzoanthamine*.* An effective administration of MMP-1 inhibitors is beginning a new treatment approach in the management of many diseases such as osteoporosis, autoimmune diseases and cancer.

Hence, conducting clinical trials on synthetic and natural zoanthamine marine alkaloids promises exciting in many groundbreaking experiments such as anti-osteoporosis effects of ligand 4 (zoanthamide) and ligand 10 (zooxathellamine) and their derivates (enolic-iminuim forms). The detailed description of protein-inhibitor interactions will aid in the design of compounds that selectively inhibit individual members of the MMP family. Such inhibitors will be useful in examining the function of MMPs in pathological processes.

## Abbreviation

MD: Molecular dynamics; RMSD: Root mean square deviation; MM-PB/GBSA: Molecular mechanics Poisson-Boltzmann/General Born surface area, DFT: density functional theory, B3LYP: Becke, three-parameter, Lee-Yang-Parr, RESP: Restrained electrostatic surface potential
